# Association of *IRGM* Gene Mutations with Inflammatory Bowel Disease in the Indian Population

**DOI:** 10.1371/journal.pone.0106863

**Published:** 2014-09-05

**Authors:** Kirankumar Baskaran, Srinivasan Pugazhendhi, Balakrishnan S. Ramakrishna

**Affiliations:** 1 Wellcome Trust Research Laboratory, Christian Medical College, Vellore, India; 2 SRM Institutes for Medical Science, Vadapalani, Chennai, India; INSERM, France

## Abstract

**Background:**

Mutations in the *IRGM* gene have been associated with Crohn's disease in several populations but have not been explored in Indian patients with this disease. This study examined the association of *IRGM* mutations with ulcerative colitis and Crohn's disease in Indian patients with inflammatory bowel disease.

**Methods:**

The *IRGM* gene was amplified in four segments and Sanger-sequenced in 101 participants (42 Crohn's disease, 39 ulcerative colitis, and 20 healthy controls). Ten single nucleotide polymorphisms (SNP) were genotyped in 1200 participants (352 Crohn's disease, 400 ulcerative colitis, and 448 healthy controls) using Sequenom MassARRAY iPLEX. Disease associations were evaluated for each of the ten SNPs.

**Results:**

Thirty one mutations were identified in the *IRGM* gene, of which two had not hitherto been reported (150226250- ss947429272 & 150227858- ss947429273). Ten SNPs (6 from the above and 4 from the literature) were evaluated. Significant associations with Crohn's disease were noted with the T allele of rs1000113 (OR 1.46, 95% CI 1.12–1.90), T allele of rs9637876 (OR 1.25, 95% CI 1.005–1.561) and C allele of rs 13361189 (OR 1.33, 95% CI 1.07–1.669). Two SNPs – rs11747270 and rs180802994 – did not exhibit Hardy-Weinberg equilibrium but were associated with both Crohn's disease and ulcerative colitis in this population. The remaining SNPs did not show significant associations with either Crohn's disease or ulcerative colitis.

**Conclusions:**

Association of *IRGM* gene SNPs with Crohn's disease is reported for the first time in Indian patients. We also report, for the first time, an association of rs 9637876 in the *IRGM* gene with Crohn's disease.

## Introduction

The inflammatory bowel diseases (IBD) are a group of immune mediated chronic systemic debilitating disorders of the gastrointestinal tract, which have been increasing in incidence worldwide in the last few decades [1]. IBD is considered to be the result of an aggressive T effector cell immune response to gastrointestinal luminal bacteria in genetically susceptible individuals [2]. Ever since the identification in 2001 of nucleolar oligomerization and binding domain 2 (*NOD2*) gene mutations in patients with Crohn's disease [3], [4], it is considered that the genetic factor in IBD relates to defective innate immunity. The common *NOD2* gene polymorphisms conferring susceptibility to Crohn's disease in Western populations are absent in Indian IBD patients [5], [6]. Analysis of genotype-level data from 15 genome wide association studies (GWAS) of Crohn's disease and/or ulcerative colitis has identified 163 IBD loci that met genome-wide significance thresholds [7]. Among the IBD susceptibility loci identified by GWAS, mutations in autophagy-related genes stand out prominently in several populations. Xenophagy, the process by which cells direct autophagy against intracellular pathogens and microorganisms, is an important facet of innate immunity [8]. Autophagy-related genes in which mutations have been identified as being associated with IBD include *ATG16L1* (autophagy pathway related 16 like 1), *NOD2*, and *IRGM* (immunity related GTPase 1) [8].

Single nucleotide polymorphisms (SNPs) in the *IRGM* gene have been reported to be associated with Crohn's disease [9], [10]. *IRGM*, located on chromosome 5q33.1, belongs to the immunity-related GTPases (IRG) family of genes [11]. In mice, the protein Irgm1 (the mouse ortholog of human IRGM) was found to induce autophagy, generating large autolysosomal organelles to eradicate intracellular bacteria [12]. The SNP rs 13361189 in the *IRGM* gene has been strongly associated with CD in a genome wide association scan as well as in a replication study. A 20-kb deletion polymorphism was identified that associated to both Crohn's disease and ulcerative colitis. This polymorphism, which was located immediately upstream of the *IRGM* gene, was in perfect linkage disequilibrium with rs 13361189, and constituted a haplotype that was associated with reduced gene expression of *IRGM* suggesting that this was a causal variant [13]. Small insertion/deletion polymorphisms in the promoter region of IRGM along with a microsatellite variant in the Alu sequence of exon-2 were independently associated with Crohn's disease. Associations between SNPs in the promoter region of *IRGM* and reduced *IRGM* gene expression have been confirmed in lymphocytes from Crohn's disease patients [14].

A second mechanism through which SNPs in the *IRGM* locus may influence disease is through micro RNAs. A synonymous coding variant (rs 10065172, c.313C>T) has been described in *IRGM* in which c.313C is a protective variant of *IRGM* while c.313T is the risk-associated allele. The micro RNA family miR-196 down-regulates the protective variant without altering the risk associated allele. SNP rs 10065172 alters the binding site for miR-196 and down regulates the *IRGM* protective variant thereby modulating autophagy of intracellular bacteria [10]. It has been shown that impairment of cell autophagy allows intracellular replication of adherent invasive *E. coli*, a bacterium associated with severe ileal Crohn's disease [15]. Pathogens have developed mechanisms to bypass the fusion of phagosome with lysosome and thereby translocate into cytosolic compartments, but IRGM protein interferes with this cytosolic replication and enhances degradation of these pathogens. A particular homozygous *IRGM* variant has been associated with increased expression of IRGM protein by causing loss of potential transcription factor binding sites, as a consequence of which autophagic degradation of translocated bacteria was facilitated [16].

There are, to date, no studies of *IRGM* gene defects in IBD patients of Indian origin. In the present study we examined whether genetic variants in the *IRGM* gene were associated with either Crohn's disease or ulcerative colitis in the Indian population.

## Methods

### Participants

This study included a total of 752 IBD patients (352 patients with CD and 400 with UC), and 448 unrelated healthy controls. Participants were recruited from patients attending the Inflammatory Bowel Diseases and Gastroenterology Clinics of the Department of Gastrointestinal Sciences in Christian Medical College, Vellore between 2003 and 2011. The diagnosis of IBD (ulcerative colitis and Crohn's disease) was based on standard consensus criteria in which clinical, radiological, endoscopic, and histopathological findings and response to treatment (when appropriate) were all considered [5], [17], [18]. Patients with proven intestinal or extra-intestinal tuberculosis, and those who refused consent to participate were excluded. Controls were recruited from unrelated healthy individuals attending the outpatient clinic for health check-up. In order to eliminate variations due to differences in region of origin of participants, IBD patients and controls were broadly matched for region. Patients were investigated and clinical data were recorded according to the usual clinical protocol followed in the Department.

### Sequencing of IRGM gene for mutations

Five mL samples of venous blood were collected in EDTA-coated Vacutainer tubes. DNA was isolated from whole blood using the salting out method and the yield and quality checked.

As there was no information on *IRGM* gene SNPs in the Indian population, we first sequenced the gene in 101 individuals (42 patients with Crohn's disease, 39 with ulcerative colitis and 20 healthy controls). The *IRGM* gene in its predominant form spans 2.2 kb and is composed of two exons [19], [20]. We amplified the gene in four overlapping segments, using forward and reverse primers (Table 1) that were either taken from the literature [16] or newly designed using the software program GeneFisher2 [21]. The entire 5′UTR including exon 1 of 698 bp, ERV9 LTR and promoter, the intervening intron, Alu segment, the open reading frame (ORF) of 546 coding bp and 250 bp of the 3′ region distal of the ORF of the IRGM gene were sequenced. The target sequencing regions were amplified by polymerase chain reaction (PCR) in a reaction mix containing 1x Taq DNA Polymerase Master Mix Red (Catalog No. 190303, Ampliqon A/S, Odense, Denmark) which was composed of 0.2 units/µl Ampliqon Taq DNA Polymerase, the NH4^+^ buffer system, 0.4 mM dNTPs and 1.5 mM magnesium chloride, 200 nM of forward and reverse primer. 1.6 M Betaine was used as an additive to prevent non-specific amplifications. The PCR was carried out in a gradient thermal cycler (Mastercycler Gradient, Eppendorf, Hamburg, Germany). The PCR conditions were as follows: initial denaturation at 95°C for 5 minutes, followed by 40 cycles of denaturation at 94°C for 30 seconds, annealing at 52°C for 30 seconds, initial extension at 72°C for 30 seconds, and final extension at 72°C for 10 minutes. The PCR products were subjected to agarose gel electrophoresis and then purified using a PureLink PCR Purification Kit (Catalog No. K3100-02, Life Technologies, Carlsbad, CA, USA). The purified amplicons were quantitated using a NanoDrop 2000c UV-Vis spectrophotometer (Thermo Fisher Scientific, Wilmington, DE, USA).

**Table 1:** Primers and products in PCR amplification of *IRGM* gene.

S.No	Forward primer	Reverse primer	Chromosome position	Product size (bp)
1	GCACCCTGTCAAAATGGA	GGAGCACACGTGCAGA	150225954–150226769	816
2	CCTTGAAAAAGAGCAGAGCATT	TAGCATCCCCAGCCCTCA	150226611–150227208	598
3	TTGCTCCCTGAAGAAATGTG	CTCAACATTCATGGCTTCCA	150227108–150227706	599
4	GGCCAGCATTGGGGTA	TTTTAGCACCTGGTGGA	150227659–150228470	812

52°C was the annealing temperature for all four overlapping PCRs. Primers 2 & 3 were chosen from a previously published article (17) and primers 1 & 4 were newly designed using GeneFisher2 (20).

Bidirectional sequencing of the amplicons was done using BigDye Terminator chemistry in a 3730 xl DNA Analyzer (Applied Biosystems, Foster City, CA, USA) at Macrogen, Seoul, Korea. The sequences were trimmed, checked for quality control and aligned against reference sequences, and SNPS identified using novoSNP 3.0.1 software [22].

Genotyping of ten *IRGM* polymorphisms (Table 2) was performed using the Sequenom-MassARRAY iPLEX strategy at NxGenBio Life Sciences (New Delhi), in 752 patients and 448 controls. In this assay, amplification primers were designed for locus specific PCR at the SNP of interest followed by locus specific primer extension (iPLEX) with single complementary mass-modified base. The primer sequences used for PCR and extension are listed in Table S1. SNPs or small insertion/deletion polymorphisms were identified in the amplified DNA by incubation with mass-modified dideoxynucleotide terminators followed by matrix-assisted laser desorption ionization–time-of-flight mass spectrometry to identify the mass [23]. Genotype allocation was done automatically, based on the mass, by the program MassARRAY TYPER (Sequenom).

**Table 2:** SNPs identified by sequencing of the *IRGM* gene in Indians.

Position	Variant	rs #	Localization
150226250	C/G	ss947429272	5′UTR
150227858	A/G	ss947429273	Exon 2
150226722	A/C	rs10059011	5′ UTR
150227736	G/C	rs180802994	Exon 2
150227966	C/A	rs72553867	Exon 2
150227425	C/T	rs9637876	Alu sequence
150226034	insTGGG	rs141639161	Promoter
150226074	A/G	rs11741515	5′near gene
150226095	A/G	rs12654043	Exon 1
150226108	T/C	rs112017023	5′UTR
150226141	G/A	rs111719118	5′UTR
150226172	C/T	rs113199847	5′UTR
150226204	T/C	rs35898555	5′UTR
150226230	A/G	rs34156253	5′UTR
150226244	G/T	rs58398445	5′UTR
150226253	Del G	rs33993564	5′UTR
150226261	A/G	rs191742304	5′UTR
150226263	G/A	rs61270113	5′UTR
150226292	C/T	rs11748151	5′UTR
150226323	C/T	rs12658239	5′UTR
150226345	C/T	rs11748158	5′UTR
150226385	A/G	rs10058821	5′UTR
150226424	T/C	rs10051804	5′UTR
150226586	A/G	rs10058943	5′UTR
150226639	T/C	rs10051924	5′UTR
150226778	A/C	rs77724219	5′UTR
150226899	T/C	rs17111376	intron
150227382	insGTTT	rs60800371	Alu sequence
150227615	G/A	rs9637870	Alu sequence
150227998	C/T	rs10065172	Coding region
150228318	G/A	rs7705542	3′ near gene

### Ethics statement

Informed written consent was obtained from all participants for the genetic analyses. The consent forms and the protocol were approved by the Institutional Review Board of the Christian Medical College, Vellore.

### Statistics

To determine association of *IRGM* SNPs with IBD susceptibility, comparison of allele and genotype distributions among cases and controls was done using the PLINK v. 1.07 (website: http://pngu.mgh.harvard.edu/purcell/plink/) whole genome data analysis toolset [24]. Odds ratios (ORs), 95% confidence intervals (CIs) and P-values were calculated. Linkage maps were constructed using the data obtained from this study of SNPs in the *IRGM* gene in each participant. LD pairwise values, haplotype structure, and haplotype frequencies were determined using the Haploview software v. 4.2 [25]. Significance of difference between groups was analysed using Chi square test. The frequency distribution of the SNPs in this study was compared with that in other populations using HaploReg v2 (http://www.broadinstitute.org/mammals/haploreg/haploreg.php.) [26]. Multifactor dimensionality reduction analysis was done using mdr_3.0.2 (http://sourceforge.net/projects/mdr/) [27] to determine whether there were gene-gene interactions between *IRGM* and *NOD2* using data from this study and from our previous study on the *NOD2* gene in the same population.

## Results

### SNPs in the IRGM gene

Sequencing of the entire *IRGM* gene in 101 individuals revealed 31 SNPs in the gene which are listed in Table 3. Twenty nine of these SNPs are already described in the literature. Two novel variants were identified in our study and were detected in at least two different DNA samples. These variants have been deposited in the NCBI database and the preliminary NCBI ss numbers of the novel variants are 150226250- ss947429272 & 150227858- ss947429273.

**Table 3:** SNPs used in the genotyping study.

150226250	C/G	ss947429272	5′UTR
150227858	A/G	ss947429273	Exon 2
150226722	A/C	rs10059011	5′ UTR
150227736	G/C	rs180802994	Exon 2
150227966	C/A	rs72553867	Exon 2
150227425	C/T	rs9637876	Alu sequence
150239587	G/A	rs4958847	downstream
150240076	C/T	rs1000113	intergenic
150258867	A/G	rs11747270	intergenic
150223387	T/C	rs13361189	upstream

The first six SNPs were among those identified in the initial sequencing, and the remaining four were taken from the literature.

### IBD patients and their IRGM genotypes

Table 4 lists the characteristics of the study groups. Ten *IRGM* SNPs (including six detected above and an additional four from the literature) were selected for genotyping the entire set of cases and controls (Table 2). The basis for selecting these 10 was the previous description in the literature of their association with IBD in other populations. Direct sequencing data obtained from patients and healthy control subjects were used to create linkage disequilibrium blocks using Haploview software v. 4.2. Tagging SNPs from each block were chosen for genotyping by setting r2 value equal to one. In addition to the tagging SNPs, rs49588471,2 rs117472703 and rs10001134 lying in the downstream regions and rs133611891 in the upstream region, were selected for typing as these SNPs did not fall under sequencing region and were reported to be strongly associated with CD in previous studies [9], [28], [29], [30]. All the SNPs investigated were in Hardy Weinberg equilibrium (HWE) in cases and controls except for rs180802994 and rs11747270.

**Table 4:** Clinical characteristics of the study population.

	CD	UC	Controls
Mean Age (mean±SD)	35±13	39±16	40±13
Gender (Male/Female)	229/123	250/150	291/157
**Disease Location**			
Ileum (L1)	117	Proctitis (E1)	82
Colon (L2)	80	Left sided (E2)	104
Ileocolon (L3)	108	Pancolitis (E3)	208
Upper GI (L4)	7		
Ileocecal	28		
**Disease Behavior**			
Inflammatory (B1)	268		
Stricturing (B2)	46		
Penetrating (B3)	18		
**Surgical Intervention**	34	2	

Table 5 and Table 6 show the the allele frequencies and genotype for each SNP in controls, patients with Crohn's disease and patients with ulcerative colitis. The T allele of rs1000113, was significantly associated with Crohn's disease with odds ratio (OR) and 95% confidence intervals (CI) of 1.46 (1.12–1.90).

**Table 5:** Allele frequencies of *IRGM* SNPs in cases and controls.

Marker	Minor Allele	Controls MAF	Crohn's disease			Ulcerative colitis		
			MAF	P-value	OR(95%CI)	MAF	P-value	OR(95%CI)
rs1000113	T	0.1465	0.2009	**0.0041**	1.46(1.12–1.90)	0.1679	0.2269	1.17(0.90–1.52)
rs4958847	A	0.3116	0.3507	0.1022	1.19(0.965–1.475)	0.3274	0.4909	1.07(0.87–1.32)
rs9637876	T	0.2643	0.3103	**0.0446**	1.25(1.005–1.561)	0.2727	0.6983	1.04(0.84–1.29)
rs10059011	C	0.4315	0.437	0.8282	1.02(0.836–1.25)	0.4141	0.4735	0.93(0.76–1.13)
rs11747270	G	0.4383	0.4728	0.1710	1.14(0.941–1.402)	0.4384	0.9967	1(0.82–1.213)
rs13361189	C	0.2466	0.3043	**0.0104**	1.33(1.07–1.669)	0.2663	0.3551	1.10(0.89–1.38)
rs72553867	A	0.0651	0.0598	0.6629	0.91(0.605–1.376)	0.0726	0.5424	1.12(0.77–1.63)
ns150227858	G	0.0044	0.0057	0.7281	1.27(0.318–5.131)	0.0100	0.1758	2.24(0.67–7.49)
ns150226250	G	0.00111	0	0.3760	0	0	0.3440	0
rs180802994	C	0.0458	0.0800	**0.0046**	1.80(1.194–2.741)	0.0691	0.0394	1.54(1.01–2.34)

Statistically significant associations are highlighted.

**Table 6:** Genotype distributions of *IRGM* SNPs in cases and controls.

SNP	Genotype	Controls (%)	Crohn's Disease		Ulcerative Colitis	
			Patients	P-Value	Patients	P-Value
rs1000113	CC	328 (73)	229 (65.2)	**0.020**	277 (69)	0.444
	CT	107 (24)	103 (29.3)		110 (28)	
	TT	12 (3)	19 (5.4)		12 (3)	
rs4958847	GG	205 (47)	144 (42)	0.252	181 (46)	0.550
	GA	193 (44)	160 (46)		168 (43)	
	AA	40 (9)	41 (12)		45 (11)	
rs9637876	CC	228 (52)	158 (45.4)	0.105	203 (51)	0.876
	CT	187 (43)	164 (47.1)		170 (43)	
	TT	22 (5)	26 (7.5)		23 (6)	
rs10059011	AA	133 (30)	97 (28)	0.525	132 (33)	0.653
	AA	232 (53)	199 (57)		200 (51)	
	CC	73 (17)	53 (15)		64 (16)	
rs11747270	AA	243 (54.4)	161 (46)	**1.13×10^−6^**	206 (52)	**0.0038**
	AG	15 (3.4)	46 (13)		35 (9)	
	GG	188 (42.2)	142 (41)		157 (39)	
rs13361189	TT	245 (56)	165 (47)	**0.031**	212 (53)	0.570
	TC	173 (39)	157 (45)		160 (40)	
	CC	22 (5)	28 (8)		26 (7)	
rs72553867	CC	389 (87.4)	310 (88.3)	0.662	344 (86)	0.780
	CA	54 (12.1)	40 (11.4)		52 (13)	
	AA	2 (0.5)	1 (0.3)		3 (1)	
ns150226250	CC	446 (99.8)	349 (100)	0.376	399 (100)	0.344
	CG	1 (0.2)	0		0	
	GG	0	0		0	
ns150227858	AA	443 (99)	346 (98.9)	0.727	392 (98)	0.174
	AG	4 (1)	4 (1.1)		8 (2)	
	GG	0	0		0	
rs180802994	GG	425 (95)	316 (90.3)	**0.006**	364 (91.4)	**0.016**
	GC	3 (1)	12 (3.4)		13 (3.3)	
	CC	19 (4)	22 (6.3)		21 (5.3)	

Statistically significant associations are highlighted.

The T allele of rs9637876 was also positively associated with Crohn's disease with OR (95% CI) of 1.25 (1.005–1.561).

The C allele of rs13361189 was associated with Crohn's disease with OR (95% CI) of 1.33 (1.07–1.669). In keeping with this, the TT genotype of rs13361189 showed a protective association with Crohn's disease (P = 0.0310).

The C allelotype of rs180802994 was associated with both Crohn's disease and ulcerative colitis with OR (CI) of 1.80 (1.194–2.741) and 1.54 (1.01–2.34) respectively. The AA genotype of rs11747270 showed a protective association with both Crohn's disease and ulcerative colitis. However, genotype frequencies of these two polymorphisms did not show evidence of HWE and therefore the validity of these associations is not clear.

The allele and genotype frequencies of rest of the *IRGM* SNPs did not show a significant difference between Crohn's disease patients and controls.

**Figure 1:**
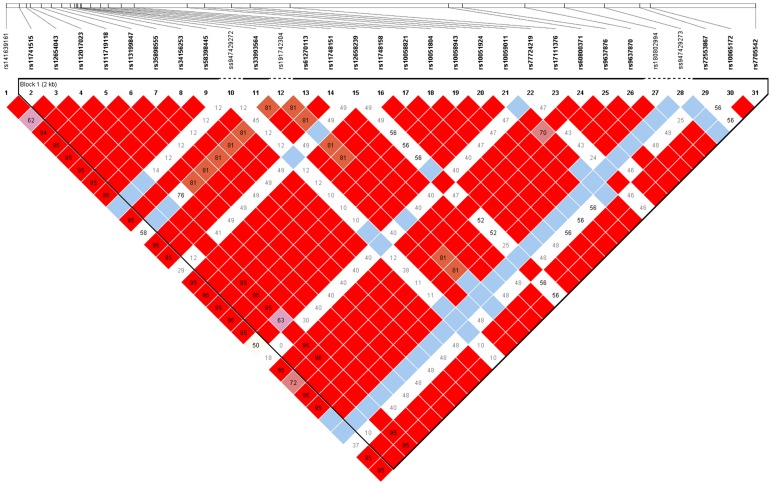
Linkage pattern between IRGM SNPs. rs13361189, rs10059011, rs9637876, rs72553867, rs4958847 and rs1000113 were in tight linkage dysequilibrium and were organised in a single haplotype block (D′ = 1).

The entire dataset from this study (SNPs shown in Table 2) were used to reconstruct a linkage map representing the chromosomal *IRGM* gene association in the Indian population (Figure 1). The following SNPs - rs13361189, rs10059011 & rs9637876, rs72553867, rs4958847, rs1000113 - were in tight linkage dysequilibrium and were organised in a single haplotype block (D′ = 1). Of the 5 haplotypes formed from 6 SNPs, 2 haplotypes (C-C-T-C-A-T, T-C-C-C-G-C) were statistically significantly associated with Crohn's disease (Table 7, P = 0.0098 and 0.0121 respectively).

**Table 7:** Haplotype frequency in cases and controls.

BLOCK 1	Control Frequency	CD Frequency	OR (95%CI)	P-VALUE	UC Frequency	OR (95%CI)	P-VALUE
T-A-C-C-G-C	0.50	0.50	1.01 (0.83–1.23)	0.8862	0.51	1.06 (0.88–1.29)	0.4924
**C-C-T-C-A-T**	0.14	0.19	1.42 (1.08–1.84)	**0.0098**	0.16	1.17 (0.90–1.52)	0.237
**T-C-C-C-G-C**	0.17	0.12	0.69 (0.52–0.92)	**0.0121**	0.14	0.78 (0.60–1.02)	0.071
C-C-T-C-A-C	0.09	0.09	0.91 (0.64–1.28)	0.5671	0.08	0.89 (0.64–1.24)	0.504
T-A-C-A-A-C	0.06	0.05	0.83 (0.54–1.28)	0.3609	0.06	1.02 (0.69–1.52)	0.896

Haplotypes indicated in Bold exhibited plausible association with CD. Rest of the haplotypes did not show any association with CD. None of these haplotypes was associated with UC.

Evaluation of genotype-phenotype correlations in patients with Crohn's disease and in ulcerative colitis did not show any significant association of genotype with disease phenotype (Tables S2 and S3).

Multifactor dimensionality reduction analysis did not show evidence for any gene-gene interaction between *IRGM* and *NOD2* in this population (Figure S1). The *IRGM* polymorphisms were associated with Crohn's disease and the *NOD2* polymorphisms were associated with ulcerative colitis.

## Discussion

The IRGM protein plays a pivotal role in inducing autophagy and eradication of intracellular mycobacteria. Persistence of intracellular bacteria is facilitated by the reduced function or activity of the *IRGM* gene, playing a significant role in Crohn's disease pathogenesis. The autophagy-stimulating *IRGM* gene has been recognised as an independent major Crohn's disease susceptibility locus in several studies [9], [13], [14]. In the present study, the association between genetic variants in the *IRGM* gene and IBD susceptibility was explored in the Indian population. The SNPs rs1000113, rs 9637876 and rs 13361189 in the *IRGM* gene were found to be associated significantly with Crohn's disease.

In accordance with our results, the SNP rs1000113 has been associated with Crohn's disease in both German and Ashkenazi Jewish populations [31], [32]. Consistent with our finding where the OR for Crohn's disease association with the minor T allele of rs1000113 was 1.46, a previous study in an Italian population of IBD patients indicated rs1000113 as a CD susceptibility locus with OR of 1.62 (Confidence interval 1.29–2.03) [28]. rs1000113 was also very strongly associated with Crohn's disease in the Wellcome Trust case control consortium study with a P-value of 5.10×10^−8^ [29]. Functional studies are further needed to elucidate the exact role of rs1000113 in the pathogenesis of CD.

The SNP rs13361189 has also been associated with Crohn's disease in other populations [31], [32]. Analyzing 37 SNPs at 31 distinct loci, Parkes et al obtained strongest replication for SNP rs13361189 (P = 6.6×10^−4^). These investigators also identified a significant association between Crohn's disease and an exonic synonymous SNP - rs10065172 – which was in near–perfect linkage disequilibrium with rs13361189, suggesting that causal variants may lie in regulatory sequences in LD with the associated SNPs [9]. In studies where five *IRGM* variants (promoter indel, microsatellite variant, upstream CNV, rs13361189 & rs10065172) were found to be associated with Crohn's disease in Europeans, the most significant association was with rs13361189 with a p value of 3.73×10^−9^. An association between the risk allele of rs13361189 and reduced IRGM expression has been confirmed in Africans [14]. The positive association of rs13361189 with CD has also been reported in Eastern European (OR 1.36) and Spanish (OR 1.34) populations [33], [34]. Population-specific genetic differences are evidenced by the lack of association of variant rs13361189 with Crohn's disease in Japanese patients [14], [35].

We report, for the first time, an association of rs 9637876 in the *IRGM* gene with Crohn's disease. This SNP has not previously been examined in Crohn's disease. However, studies in Ghanian patients and controls indicated that rs9637876 was associated with decreased susceptibility to tuberculosis caused by *Mycobacterium tuberculosis* but not that caused by *M. africanum* or *M. bovis* [16].

rs10059011, which was identified as an *IRGM* variant that contributes to protection against tuberculosis [16], was present in our population also, but was not associated with IBD, either in causal manner or as a protective association.

rs11747270 which was found to have the strongest association (P = 6.36×10^−11^) with Crohn's disease in a meta-analysis of three GWAS [30] showed an association with both Crohn's disease and ulcerative colitis in our patients, but the lack of HWE in our study population with respect to this SNP indicates that this association remains unconfirmed. The minor allele of rs11747270 was also significantly associated with Crohn's disease in Ashkenazi Jews (OR 1.48), and with ulcerative colitis in Germans [31], [32]. There is also evidence for an association between the risk allele (G) of rs11747270 and increased anti–flagellin seropositivity suggesting that defects in autophagy might influence development of anti-flagellin antibody [36].

SNPs rs72553867, rs4958847 were associated with Crohn's disease in Korean, Italian and US populations [28], [37], [38]. These variants did not show any significant association with IBD in our study. It is interesting that ulcerative colitis was not associated with any of these *IRGM* variants.

The functional connotations of the three significantly associated *IRGM* SNPs - rs1000113, rs 9637876 and rs 13361189 – remain to be elucidated. Presently there does not appear to be any experimental evidence identifying the cellular consequences of these SNPs.

Table S4 compares genotype frequencies of the various *IRGM* SNPs between the present study in the Indian population and African, Asian, American and European populations. The frequencies in Indians resemble frequencies in Asian and African populations, specifically showing higher frequency of SNPs rs9637876 and rs11747270 compared to American and European populations.

Analyzing data presented here in this study and comparing it with data on *NOD2* SNPs obtained in this same population in a previous study, we did not find any evidence for gene-gene interaction between these two genes in terms of their association with inflammatory bowel disease, Crohn's disease or ulcerative colitis.

Genotype frequencies of two SNPs - rs11747270 and rs180802994 - showed deviations from HWE in our population with a preponderance of homozygotes in both controls and cases. This may potentially be explained by the phenomenon of ‘population stratification’, where mate selection in a population is restricted to members of one particular subgroup within that population, as a consequence of which there is an excess of homozygotes and a corresponding deficiency of heterozygotes compared with random mating in the population as a whole. Consanguinity and inbreeding are other explanations postulated for deviations in HWE. Even though both these SNPs were statistically associated with both Crohn's disease and ulcerative colitis in our patients, the deviation from HWE increases the possibility of a Type 1 error with false positive association of marker and disease even if they are not genetically linked [39]. It is unfortunate that data on frequency of rs180802994 variant in other populations are not available so far.

In summary this study identified rs13361189, rs1000113 and rs9637876 as significant SNPs in *IRGM* to be associated with Crohn's disease, but not ulcerative colitis, susceptibility in an Indian population. Functional analyses will be necessary to understand the relationship between these candidate causal variants in the *IRGM* gene and CD pathogenesis.

## Supporting Information

Figure S1 **Statistical model for association between polymorphisms in the** ***NOD2*** **gene (rs2111235 and rs2066843) and** ***IRGM*** **gene (rs11747270 and rs1000113) and IBD overall, Crohn's disease (CD) and ulcerative colitis (UC).** Representative examples of the analysis are shown. The distribution of cases (left bars) and controls (right bars) is shown for each multilocus gene combination. Dark shaded cells are high risk for disease and light shaded cells are low risk for disease.
(DOCX)

Table S1 **Primer sequences (for PCR and extension) used for the iPLEX reaction.**
(DOCX)

Table S2 **Genotype-phenotype correlation in patients with Crohn's disease. Values shown are patient numbers.**
(DOCX)

Table S3 **Genotype-phenotype associations in patients with ulcerative colitis. Values shown are patient numbers.**
(DOCX)

Table S4 **Frequency distribution of** ***IRGM*** **gene SNPs among different populations.**
(DOCX)
