# Supplementary material for: Association of IRGM Gene Mutations with Inflammatory Bowel Disease in the Indian Population
Source: PLoS One. 2014 Sep 5;9(9):e106863. doi: 10.1371/journal.pone.0106863 (PMC4156415; doi:10.1371/journal.pone.0106863)
Supplement: Figure S1 — Statistical model for association between polymorphisms in the NOD2 gene (rs2111235 and rs2066843) and IRGM gene (rs11747270 and rs1000113) and IBD overall, Crohn's disease (CD) and ulcerative colitis (UC). Representative examples of the analysis are shown. The distribution of cases (left bars) and controls (right bars) is shown for each multilocus gene combination. Dark shaded cells are high risk for disease and light shaded cells are low risk for disease. (DOCX) [file pone.0106863.s001.docx]

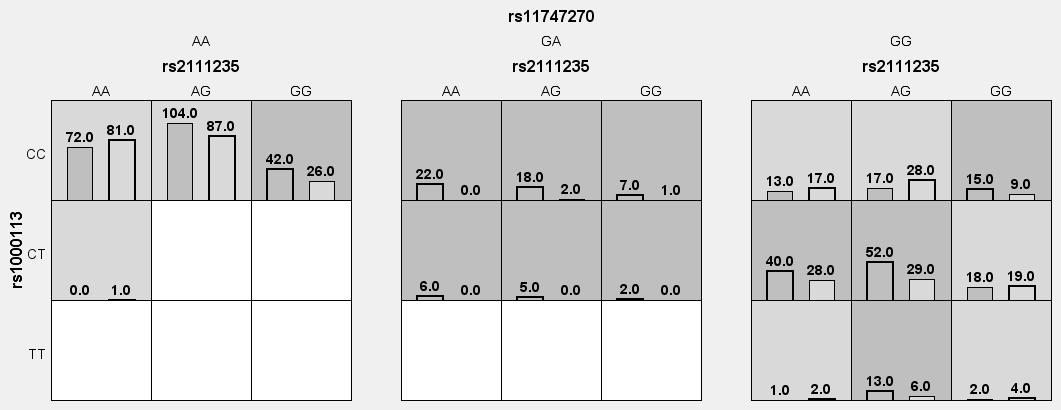


IBD: The cross validation consistency was 4/10
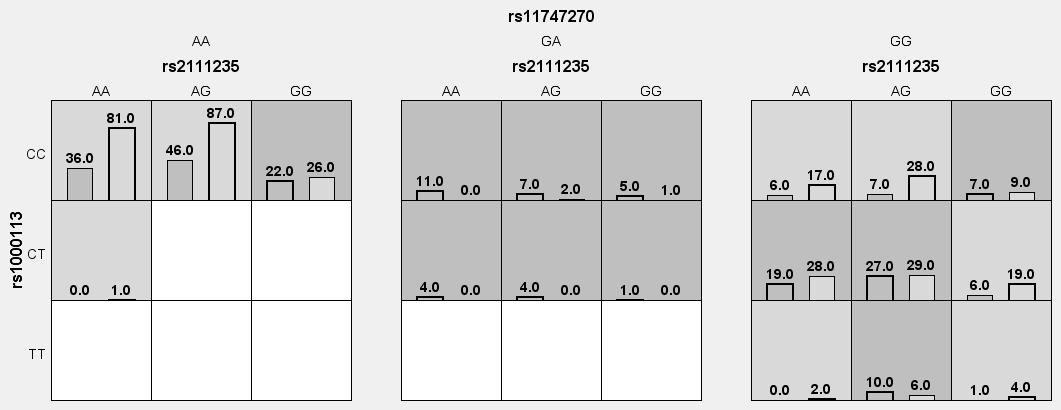


CD: The cross validation consistency was 8/10


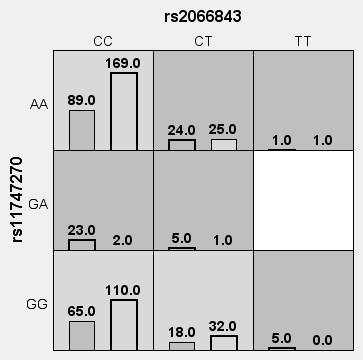


UC : The cross validation consistency was 6/10

**Supplementary Figure 1**. Statistical model for association between polymorphisms in the *NOD2* gene (rs2111235 and rs2066843) and *IRGM* gene (rs11747270 and rs1000113) and IBD overall, Crohn’s disease (CD) and ulcerative colitis (UC). Representative examples of the analysis are shown. The distribution of cases (left bars) and controls (right bars) is shown for each multilocus gene combination. Dark shaded cells are high risk for disease and light shaded cells are low risk for disease.
