# Supplementary material for: Association of IRGM Gene Mutations with Inflammatory Bowel Disease in the Indian Population
Source: PLoS One. 2014 Sep 5;9(9):e106863. doi: 10.1371/journal.pone.0106863 (PMC4156415; doi:10.1371/journal.pone.0106863)
Supplement: Table S1 — Primer sequences (for PCR and extension) used for the iPLEX reaction. (DOCX) [file pone.0106863.s002.docx]

**Supplemental Table 1.** Primer sequences (for PCR and extension) for iPLEX

| SNP | PRIMER 1 | PRIMER 2 | EXTENSION PRIMER |
| --- | --- | --- | --- |
| rs1000113 | ACGTTGGATGTGGTTGCTCAGGAAGTAAAG | ACGTTGGATGTATACTTGATTGCAATGCCC | GCAATGCCCATGTGTA |
| rs4958847 | ACGTTGGATGCTATTTTAGGGAGTAGTGC | ACGTTGGATGCTCTCACTGGGAGAAGCTTT | ATAGATTTCATTGCCCAATAT |
| rs9637876 | ACGTTGGATGAACCCTGGACGCAGATATTG | ACGTTGGATGTGAGATGGAGTCTTGCTCTG | GCTGGAGTGCAATGG |
| rs10059011 | ACGTTGGATGCAGTCTTAATGGAAGCTCGG | ACGTTGGATGCGTGCAGAGCAGTACTGAAA | TGCTGCAATATACGTGAAG |
| rs11747270 | ACGTTGGATGGGCTTACTTGCTTCCATCAG | ACGTTGGATGTGACACTGGTGTTTCAATGC | TGTTTCAATGCACATTTATGTAA |
| rs13361189 | ACGTTGGATGCCTTTCTAAACTGTACCCGC | ACGTTGGATGTGTCGTACCCAAGCAGAGTG | AAGCAGAGTGTGCTTGAAAAT |
| rs72553867 | ACGTTGGATGCAAATGTGGTGTTGTGGGAC | ACGTTGGATGACCGGTTGAACTGCATTTCC | cctcGTGGTGGCAGACCCT |
| n150227858 | ACGTTGGATGATGTCCACCTTCATCAGTGC | ACGTTGGATGGGTAGCTTTTACCAGCTCAG | GAGGCCTTACCCTCA |
| n150226250 | ACGTTGGATGAGTTACAGCTCTTCAGGCGG | ACGTTGGATGAGCATCACTCCTGAAACCTG | TGTGAAACAACGAACCC |
| rs180802994 | ACGTTGGATGGTGTCCTGGACACTATCTTC | ACGTTGGATGTGAGAAAGCCTCAGCAGATG | GATGGGAACTTGCCAGA |
