# Supplementary material for: Association of IRGM Gene Mutations with Inflammatory Bowel Disease in the Indian Population
Source: PLoS One. 2014 Sep 5;9(9):e106863. doi: 10.1371/journal.pone.0106863 (PMC4156415; doi:10.1371/journal.pone.0106863)
Supplement: Table S2 — Genotype-phenotype correlation in patients with Crohn's disease. Values shown are patient numbers. (DOCX) [file pone.0106863.s003.docx]

**Supplemental Table 2.**  Genotype-phenotype correlation in patients with Crohn’s disease and ulcerative colitis.

| **SNP** | **Genotype** | **Crohn’s Disease** | | | | **P Value** |
| --- | --- | --- | --- | --- | --- | --- |
|  |  | **Ileal** | **Colonic** | **Ileocolonic** | **Upper GI** |  |
| rs1000113 | CC | 72 | 48 | 85 | 7 | 0.282 |
|  | CT | 34 | 24 | 30 | 5 |  |
|  | TT | 9 | 1 | 8 | 0 |  |
| rs13361189 | TT | 48 | 35 | 64 | 5 | 0.244 |
|  | TC | 56 | 35 | 46 | 7 |  |
|  | CC | 11 | 3 | 12 | 0 |  |
| rs9637876 | CC | 47 | 36 | 60 | 4 | 0.449 |
|  | CT | 58 | 34 | 51 | 8 |  |
|  | TT | 10 | 3 | 11 | 0 |  |
| rs4958847 | GG | 42 | 29 | 57 | 5 | 0.268 |
|  | GA | 54 | 37 | 49 | 7 |  |
|  | AA | 17 | 5 | 16 | 0 |  |
| rs10059011 | AA | 31 | 23 | 35 | 2 | 0.204 |
|  | AC | 61 | 45 | 65 | 9 |  |
|  | CC | 22 | 5 | 22 | 1 |  |
| rs72553867 | CC | 99 | 65 | 110 | 11 | 0.731 |
|  | CA | 16 | 8 | 12 | 1 |  |
|  | AA | 0 | 0 | 1 | 0 |  |
| ns150226250 | CC | 114 | 73 | 122 | 12 | NA |
|  | CG | 0 | 0 | 0 | 0 |  |
|  | GG | 0 | 0 | 0 | 0 |  |
| ns150227858 | AA | 111 | 73 | 123 | 12 | NA |
|  | AG | 3 | 0 | 0 | 0 |  |
|  | GG | 0 | 0 | 0 | 0 |  |
| rs11747270 | AA | 46 | 35 | 62 | 5 | 0.325 |
|  | AG | 16 | 10 | 20 | 0 |  |
|  | GG | 53 | 27 | 40 | 7 |  |
| rs180802994 | GG | 100 | 67 | 111 | 11 | 0.642 |
|  | GC | 5 | 2 | 3 | 1 |  |
|  | CC | 9 | 4 | 9 | 0 |  |
