# Supplementary material for: Association of IRGM Gene Mutations with Inflammatory Bowel Disease in the Indian Population
Source: PLoS One. 2014 Sep 5;9(9):e106863. doi: 10.1371/journal.pone.0106863 (PMC4156415; doi:10.1371/journal.pone.0106863)
Supplement: Table S3 — Genotype-phenotype associations in patients with ulcerative colitis. Values shown are patient numbers. (DOCX) [file pone.0106863.s004.docx]

**Supplemental Table 2:** Genotype-phenotype associations in patients with ulcerative colitis

| **SNP** | **Genotype** | **Ulcerative Colitis** | | | **P Value** |
| --- | --- | --- | --- | --- | --- |
|  |  | **Proctitis** | **Left Sided** | **Pancolitis** |  |
| rs1000113 | CC | 37 | 69 | 148 | 0.135 |
|  | CT | 24 | 29 | 54 |  |
|  | TT | 3 | 2 | 6 |  |
| rs13361189 | TT | 25 | 51 | 117 | 0.247 |
|  | TC | 33 | 42 | 78 |  |
|  | CC | 5 | 7 | 13 |  |
| rs9637876 | CC | 23 | 48 | 113 | 0.1 |
|  | CT | 36 | 45 | 83 |  |
|  | TT | 6 | 5 | 11 |  |
| rs4958847 | GG | 23 | 40 | 104 | 0.202 |
|  | GA | 30 | 44 | 85 |  |
|  | AA | 10 | 13 | 18 |  |
| rs10059011 | AA | 12 | 34 | 75 | 0.1 |
|  | AC | 37 | 50 | 100 |  |
|  | CC | 15 | 14 | 32 |  |
| rs72553867 | CC | 55 | 82 | 187 | 0.145 |
|  | CA | 9 | 17 | 20 |  |
|  | AA | 0 | 1 | 1 |  |
| ns150226250 | CC | 64 | 100 | 208 | NA |
|  | CG | 0 | 0 | 0 |  |
|  | GG | 0 | 0 | 0 |  |
| ns150227858 | AA | 64 | 98 | 203 | NA |
|  | AG | 1 | 2 | 5 |  |
|  | GG | 0 | 0 | 0 |  |
| rs11747270 | AA | 24 | 49 | 115 | 0.08 |
|  | AG | 10 | 10 | 14 |  |
|  | GG | 29 | 41 | 79 |  |
| rs180802994 | GG | 57 | 94 | 187 | 0.12 |
|  | GC | 2 | 3 | 7 |  |
|  | CC | 6 | 1 | 14 |  |
