# Supplementary material for: Association of IRGM Gene Mutations with Inflammatory Bowel Disease in the Indian Population
Source: PLoS One. 2014 Sep 5;9(9):e106863. doi: 10.1371/journal.pone.0106863 (PMC4156415; doi:10.1371/journal.pone.0106863)
Supplement: Table S4 — Frequency distribution of IRGM gene SNPs among different populations. (DOCX) [file pone.0106863.s005.docx]

**Supplemental Table 4**. Frequency distribution of *IRGM* gene SNPs among different populations.

| **SNP** | **AFR** | **AMR** | **ASN** | **EUR** | **IND *** |
| --- | --- | --- | --- | --- | --- |
| rs1000113 | 0.23 | 0.14 | 0.41 | 0.09 | 0.2009 |
| rs4958847 | 0.48 | 0.22 | 0.63 | 0.13 | 0.3507 |
| rs9637876 | 0.31 | 0.14 | 0.44 | 0.09 | 0.3103 |
| rs10059011 | 0.52 | 0.41 | 0.45 | 0.54 | 0.437 |
| rs11747270 | 0.49 | 0.15 | 0.44 | 0.09 | 0.4728 |
| rs13361189 | 0.46 | 0.15 | 0.44 | 0.09 | 0.3043 |
| rs72553867 | 0.01 | 0.05 | 0.18 | 0.04 | 0.0598 |
| ns150227858 | NA | NA | NA | NA | 0.0057 |
| ns150226250 | NA | NA | NA | NA | 0 |
| rs180802994 | NA | NA | NA | NA | 0.08 |

*Data from present study.
